# Supplementary material for: Methodological Development of a Test for Salivary Proteome Analysis Useful in Lung Cancer Screening
Source: Int J Mol Sci. 2025 Aug 16;26(16):7924. doi: 10.3390/ijms26167924 (PMC12386888; doi:10.3390/ijms26167924)
Supplement: Supplementary file 1 [file ijms-26-07924-s001.zip › Table S1.pdf]

Label	ABBR. Name	Mw/*pI* Theor.	PMF Score [a]	Peptide matched/ peptide searched	SC[b] %	Lift (MS2) Ion parent masses (m/z)	Score [C] Tof-Tof	Peptide Sequence
M2	**IFNA1**	22.11/ 5.32	45	5	32	1108.5125<br>1247.6590<br>1464.6488	318	ISPSSCLMDR<br>SLSLSTNLQER<br>DSSAAWDEDLLDK
M3	S10A9	13.29/ 5.71	101	13	90	2175.9624<br>1455.7226	201	MHEGDEGPGHHHKPGLGEGT<br>LGHPDTLNQGEFK
M8	CYTN	16.60/ 6.73	70	11	68	1914.9344<br>1292.6633	189	QQTVGGVNYFFDVEVGR<br>ALHFAISEYNK
M11	ENOB	47.69/ 6.5	102	27	67	1166.6316<br>1380.7117<br>2296.1376	263	IGAEVYHHLK<br>GNPTVEVDLHTAK<br>SGETEDTFIADLVVGLCTGQ IK
M12	CAH6	35.45/ 6.51	94	11	25	2288.0982<br>1588.8594	221	NYPENTYYSNFISHLANIK<br>HVIEIHIVHYNSK
M19	PIGR	84.42/ 5.58	93	29	39	1228.7623<br>1539.6517<br>2114.1968	136	LVSLTLNLVTR<br>ASVDSGSSEEQGGSSR<br>LDIQGTGQLLFSVVINQLR
M21	AMY1A	58.41/ 6.47	160	21	49	1099.6721<br>1726.8031	101	LSGLLDLALGK<br>DVNDWVGPPNDNGVTK
M25	PIGR	84.42/ 5.58	40	21	43	1228.7623<br>2025.9664	227	LVSLTLNLVTR<br>QGHFYGETAAVYVAVEER
M22	CYTS	16.48/ 4.95	87	13	73	1292.6633<br>1963.9184<br>2074.0239	192	ALHFAISEYNK<br>EQTFGGVNYFFDVEVGR<br>IIPGGIYDADLNDEWVQR
M24	HS71A	70.29/ 5.48	126	19	39	1197.6949<br>1465.8121<br>1877.8949	98	DAGVIAGLNVLR<br>AQIHDLVLVGGSTR<br>CQEVISWLDANTLAEK
M29	CYTN	16.60/ 6.73	138	4	37	1914.9344<br>1292.6633	142	QQTVGGVNYFFDVEVGR<br>ALHFAISEYNK
M50	AMYP	58.41/ 6.47	160	27	49	1002.4275<br>1185.7089<br>1918.9843	295	SSDYFGNGR<br>LTGLLDLALEK<br>IAEYMNHLIDIGVAGFR
D9	ILEU	5.90/ 42.82	54	19	39	1149.5898<br>1510.7383<br>1650.8077	147	FQSLNADINK<br>LEESYTLNSDLAR<br>IPELLASGMVDNMTK
D10	ACTB	5.29/ 42.05	100	27	48	976.4482<br>1790.8919<br>2231.0648	106	AGFAGDDAPR<br>SYELPDGQVITIGNER<br>DLYANTVLSGGTTMYPGIAD
D11	ERI1	6.29/ 40.49	37	17	29	1064,4863<br>1278.6623	99	LGYAGNTEPQFIIPSCIAIK<br>FLNIQCQLSR
D13	ACTG	5.31/ 42.10	102	29	60	976.4482<br>1198.7055<br>2231.0648	211	AGFAGDDAPR<br>AVFPSIVGRPR<br>DLYANTVLSGGTTMYPGIAD
D15	SERP3	6.35/ 44.59	54	26	48	1047.5622<br>1707.8336<br>1804.9479	145	GSFVHYPLK<br>GISGQDGFYVSEAIHK<br>SILNSWGVTDLFDPLK
D16	ARP3	5.61/ 47.79	85	35	53	947.4792<br>1497.7795<br>2192.1419	307	LSEELSGGR<br>EVGIPPEQSLETAK<br>LGYAGNTEPQFIIPSCIAIK
D38	BPIA2	5.35/ 27.16	90	14	56	1134.6517<br>1101.6514<br>1907.0120	111	FVNSVINTLK<br>ISNSLILDVK<br>LEPVLHEGLETVDNTLK
D39	BPIA2	5.35/ 27.16	96	14	55	962.5517<br>900.5513	247	STVSSLLQK<br>LLNNVISK
D44	PSA5	4.74/ 26.56	52	15	58	1143.4830<br>1961.9563<br>2033.0583	226	GVNTFSPEGR<br>AIGSASEGAQSSLQEVYHK<br>LGSTAIGIQTSEGVCLAVEK
D49	GML	6.10/ 18..35	41	9	62	1599.9720<br>2004.8473	183	LLLSFASIIVSNILP<br>CHDCAVINDFNCPNIR
D51	HSBP3	5.36/ 17.06	29	4	32	1866.9993<br>1593.7867	289	DLSAVLCHDGILVVE<br>AAQSPPVDSAAETPPR

Sequence validation of selected proteins from unstimulated and stimulated saliva samples by MS using LIFT technology a: PMF Score = values are Log10 (p), where p is probability that the observed match is a random event; it is based on Swiss Prot database using the MASCOT searching program; b: SC = Sequence Coverage means the ratio of portion sequence covered by matched peptide to the full length of the protein sequence. c: Score Tof-Tof = score that results from combining PMF and MS /MS matched peptide from ion parent fragments
