# Supplementary material for: Methodological Development of a Test for Salivary Proteome Analysis Useful in Lung Cancer Screening
Source: Int J Mol Sci. 2025 Aug 16;26(16):7924. doi: 10.3390/ijms26167924 (PMC12386888; doi:10.3390/ijms26167924)
Supplement: Supplementary file 1 [file ijms-26-07924-s001.zip › Table S2.pdf]

LIST OF GENES ENCODING MATCHED AND DIFFERENTLY EXPRESSED PROTEINS REPORTED IN TABLE 8 AND INCLUDED IN THE NETWORK ELABORATED BY THE STRING DATABASE

IFNA1	*Interferon alpha-1/13; Produced by macrophages, IFN-alpha have antiviral activities. Interferon stimulates the production of two enzymes: a protein kinase and an oligoadenylate synthetase; Belongs to the alpha/beta interferon family. (189 aa)*
S100A9	*Protein S100-A9; S100A9 is a calcium- and zinc-binding protein which plays a prominent role in the regulation of inflammatory processes and immune response. It can induce neutrophil chemotaxis, adhesion, can increase the bactericidal activity of neutrophils by promoting phagocytosis via activation of SYK, PI3K/AKT, and ERK1/2 and can induce degranulation of neutrophils by a MAPK-dependent mechanism. Predominantly found as calprotectin (S100A8/A9) which has a wide plethora of intra- and extracellular functions. The intracellular functions include: facilitating leukocyte arachidonic acid [...] (114 aa)*
CST1	*Cystatin-SN; Human saliva appears to contain several cysteine proteinase inhibitors that are immunologically related to cystatin S but that differ in their specificity due to amino acid sequence differences. Cystatin SN, with a pI of 7.5, is a much better inhibitor of papain and dipeptidyl peptidase I than is cystatin S, although both inhibit ficin equally well. (141 aa)*
ENO3	*Beta-enolase; Appears to have a function in striated muscle development and regeneration; Belongs to the enolase family. (434 aa)*
CA6	*Carbonic anhydrase 6; Reversible hydration of carbon dioxide. Its role in saliva is unknown. (313 aa)*
PIGR	*Polymeric immunoglobulin receptor; This receptor binds polymeric IgA and IgM at the basolateral surface of epithelial cells. The complex is then transported across the cell to be secreted at the apical surface. During this process a cleavage occurs that separates the extracellular (known as the secretory component) from the transmembrane segment. (764 aa)*
AMY1A	*Amylase alpha 1A. (511 aa)*
CST4	*Cystatin-S; This protein strongly inhibits papain and ficin, partially inhibits stem bromelain and bovine cathepsin C, but does not inhibit porcine cathepsin B or clostripain. Papain is inhibited non- competitively. (141 aa)*
HSPA1B	*Heat shock 70 kDa protein 1A; Molecular chaperone implicated in a wide variety of cellular processes, including protection of the proteome from stress, folding and transport of newly synthesized polypeptides, activation of proteolysis of misfolded proteins and the formation and dissociation of protein complexes. Plays a pivotal role in the protein quality control system, ensuring the correct folding of proteins, the re-folding of misfolded proteins and controlling the targeting of proteins for subsequent degradation. This is achieved through cycles of ATP binding, ATP hydrolysis and AD [...] (641 aa)*
AMY2A	*Pancreatic alpha-amylase; Amylase alpha 2A. (511 aa)*
SERPINB1	*Leukocyte elastase inhibitor; Neutrophil serine protease inhibitor that plays an essential role in the regulation of the innate immune response, inflammation and cellular homeostasis. Acts primarily to protect the cell from proteases released in the cytoplasm during stress or infection. These proteases are important in killing microbes but when released from granules, these potent enzymes also destroy host proteins and contribute to mortality. Regulates the activity of the neutrophil proteases elastase, cathepsin G, proteinase-3, chymase, chymotrypsin, and kallikrein-3. Acts also as a [...] (379 aa)*

ACTR3	*Actin-related protein 3; ATP-binding component of the Arp2/3 complex, a multiprotein complex that mediates actin polymerization upon stimulation by nucleation-promoting factor (NPF). The Arp2/3 complex mediates the formation of branched actin networks in the cytoplasm, providing the force for cell motility. Seems to contact the pointed end of the daughter actin filament. In addition to its role in the cytoplasmic cytoskeleton, the Arp2/3 complex also promotes actin polymerization in the nucleus, thereby regulating gene transcription and repair of damaged DNA. The Arp2/3 complex promote [...] (418 aa)*
BPIFA2	*BPI fold-containing family A member 2; Has strong antibacterial activity against P. aeruginosa. Belongs to the BPI/LBP/Plunc superfamily. Plunc family. (249 aa)*
PSMA5	*Proteasome subunit alpha type-5; Component of the 20S core proteasome complex involved in the proteolytic degradation of most intracellular proteins. This complex plays numerous essential roles within the cell by associating with different regulatory particles. Associated with two 19S regulatory particles, forms the 26S proteasome and thus participates in the ATP- dependent degradation of ubiquitinated proteins. The 26S proteasome plays a key role in the maintenance of protein homeostasis by removing misfolded or damaged proteins that could impair cellular functions, and by removing pr [...] (241 aa)*
GML	*Glycosyl-phosphatidylinositol-anchored molecule-like protein; May play a role in the apoptotic pathway or cell-cycle regulation induced by p53/TP53 after DNA damage. (158 aa)*
HSPB3	*Heat shock protein beta-3; Inhibitor of actin polymerization. (150 aa)*
ACTB	*Actin, cytoplasmic 1, N-terminally processed; Actin is a highly conserved protein that polymerizes to produce filaments that form cross-linked networks in the cytoplasm of cells. Actin exists in both monomeric (G-actin) and polymeric (F-actin) forms, both forms playing key functions, such as cell motility and contraction. In addition to their role in the cytoplasmic cytoskeleton, G- and F-actin also localize in the nucleus, and regulate gene transcription and motility and repair of damaged DNA. (375 aa)*
ACTG1	*Actin, cytoplasmic 2, N-terminally processed; Actins are highly conserved proteins that are involved in various types of cell motility and are ubiquitously expressed in all eukaryotic cells. (375 aa)*
ERI1	*3'-5' exoribonuclease 1; RNA exonuclease that binds to the 3'-end of histone mRNAs and degrades them, suggesting that it plays an essential role in histone mRNA decay after replication. A 2' and 3'-hydroxyl groups at the last nucleotide of the histone 3'-end is required for efficient degradation of RNA substrates. Also able to degrade the 3'-overhangs of short interfering RNAs (siRNAs) in vitro, suggesting a possible role as regulator of RNA interference (RNAi). Requires for binding the 5'-ACCCA-3' sequence present in stem-loop structure. Able to bind other mRNAs. Required for 5.8S rR [...] (349 aa)*
SERPINE3	*Serpin E3; Probable serine protease inhibitor. (424 aa)*
