# Supplementary material for: Methodological Development of a Test for Salivary Proteome Analysis Useful in Lung Cancer Screening
Source: Int J Mol Sci. 2025 Aug 16;26(16):7924. doi: 10.3390/ijms26167924 (PMC12386888; doi:10.3390/ijms26167924)
Supplement: Supplementary file 1 [file ijms-26-07924-s001.zip › Table S3. Informed consent .pdf]

Summary of the informed consent reported below:

The first lines contain the patient's personal data

In the next paragraph, the patient is asked to report which doctor he/she interviewed, whether the options for the processing of sensitive data were clear and whether the patient therefore consents to the collection and storage of the samples thus obtained

In the third paragraph, it is explained for what purpose the samples collected will be used (if rendered anonymous)

In the fourth paragraph, if they were not rendered anonymous, it is asked whether the patient consents to the processing of sensitive data relating to the investigations

In the fifth paragraph, it is asked whether he/she consents to the processing of molecular data (genomic and proteomic) resulting from the study of the samples obtained and whether, if important data for his/her health were derived from them, he/she consents to receiving information capable of providing a benefit in terms of therapy or prevention and whether members of his/her same genetic line can receive such information

In the last lines, in addition to the date, the patient must add his/her signature (or that of his/her legal representative)

Il/la sottoscritto/a____

□ Paziente interessato

□ Legale rappresentante del paziente: Sig./Sig.ra____

dichiara che durante il colloquio con il/la ____:<br>▪ mi sono state verbalmente illustrate in modo chiaro tutte le varie opzioni inerenti il trattamento dei dati sensibili e genomici/proteomici ed utilizzo dei campioni biologici, per il perseguimento di obiettivi di ricerca in ambito scientifico;<br>▪ mi è stato concesso il tempo necessario per formulare domande, approfondire eventuali dubbi;<br>▪ mi è stato sottolineato che le decisioni da me prese saranno considerate valide per tutti i campioni biologici che mi saranno prelevati in relazione ad una specifica patologia, in un unico momento o in momenti differenti nel corso di ulteriori ricoveri/trattamenti, e fino ad eventuale revoca o rettifica che potrò manifestare in qualsiasi momento.<br>In relazione a quanto sopra dichiarato acconsente alla conservazione/stoccaggio dei campioni biologici prelevati nel corso del trattamento diagnostico terapeutico e/o specificatamente acquisiti, e all'esecuzione sui medesimi, di indagini a scopo di ricerca senza che possano configurarsi attese di natura economica in relazione alle conoscenze acquisite:	
**1. solo se resi anonimi** (e conseguentemente se gli obiettivi delle indagini sono compatibili con tale scelta), consapevole che in tal modo non potrà venire a conoscenza di risultati utili che ne derivassero.<br>**1.1** Le ricerche perseguiranno obiettivi così riassumibili:<br>- definire i meccanismi molecolari che determinano lo sviluppo dei tumori;<br>- identificare nuovi marcatori molecolari per effettuare una diagnosi precoce, predire l'evoluzione naturale della malattia (prognosi) e la risposta alla terapia;<br>**1.2** acconsente alle medesime condizioni di cui al punto 1, alla conservazione/stoccaggio dei campioni biologici prelevati nel corso del trattamento diagnostico terapeutico ed all'esecuzione sui medesimi, di ulteriori indagini a scopi di ricerca i cui obiettivi non sono ancora noti allo stato della attuali conoscenze ma che possiamo presupporre alla luce dei costanti sviluppi tecnologici e scientifici;	□ si □ no
**2. non resi anonimi ma comunque protetti da un sistema di codifica**	
**2.1** acconsente al trattamento dei **dati sensibili** inerenti le indagini di cui al punto 2;	□ si □ no
**2.1.1** acconsente al perseguimento dei seguenti obiettivi delle ricerche:<br>- definire i meccanismi molecolari che determinano lo sviluppo dei tumori;<br>- identificare nuovi marcatori molecolari per effettuare una diagnosi precoce, predire l'evoluzione naturale della malattia (prognosi) e la risposta alla terapia;	□ si □ no
**2.1.2** acconsente alle medesime condizioni di cui al punto 2, alla conservazione/stoccaggio dei campioni biologici prelevatemi nel corso del trattamento diagnostico terapeutico e/o specificatamente acquisiti, e all'esecuzione sui medesimi di indagini a scopi di ricerca i cui obiettivi non sono ancora noti allo stato delle attuali conoscenze ma che possiamo presupporre alla luce dei costanti sviluppi tecnologici e scientifici;	□ si □ no
**2.1.3** acconsente a ricevere informazioni, qualora dalle indagini effettuate per fini di ricerca in ambito scientifico, ne conseguano informazioni, anche inattese, in grado di arrecare un beneficio concreto e diretto in termini di terapia o di prevenzione o in funzione di consapevoli scelte riproduttive;	□ si □ no
**2.2** acconsente al trattamento dei **dati molecolari (genomici/proteomici)** inerenti le indagini di cui al punto 2;	□ si □ no
**2.2.1** acconsente al perseguimento dei seguenti obiettivi delle ricerche:<br>- definire i meccanismi molecolari dello sviluppo dei tumori;<br>- identificare nuovi marcatori molecolari per effettuare una diagnosi precoce, predire l'evoluzione naturale della malattia (prognosi) e la risposta alla terapia;	□ si □ no
**2.2.2** acconsente alle medesime condizioni di cui al punto 2, alla conservazione/stoccaggio dei campioni biologici prelevate nel corso del trattamento diagnostico terapeutico e/o specificatamente acquisiti, e all'esecuzione sui medesimi di indagini a scopi di ricerca i cui obiettivi non sono ancora noti allo stato delle attuali conoscenze ma che possiamo presupporre alla luce dei costanti sviluppi tecnologici e scientifici, e che pur nell'evoluzione degli obiettivi di ricerca attualmente noti, dovranno comunque essere pertinenti con la prevenzione/cura della malattia oncologica;	□ si □ no
**2.2.3** acconsente a ricevere informazioni, qualora dalle indagini effettuate per fini di ricerca in ambito scientifico, ne conseguano informazioni, anche inattese, in grado di arrecare un beneficio concreto e diretto in termini di terapia o di prevenzione o in funzione di consapevoli scelte riproduttive;	□ si □ no
**2.2.4** acconsente a che anche gli appartenenti della sua stessa linea genetica possano ricevere informazioni di cui al punto precedente, previa specifica richiesta per iscritto.	□ si □ no

Data_____/_____/______

Firma dell'interessato (o del rappresentante legale):

____
