# Supplementary figures and images for: Methodological Development of a Test for Salivary Proteome Analysis Useful in Lung Cancer Screening
Source: Int J Mol Sci. 2025 Aug 16;26(16):7924. doi: 10.3390/ijms26167924 (PMC12386888; doi:10.3390/ijms26167924)

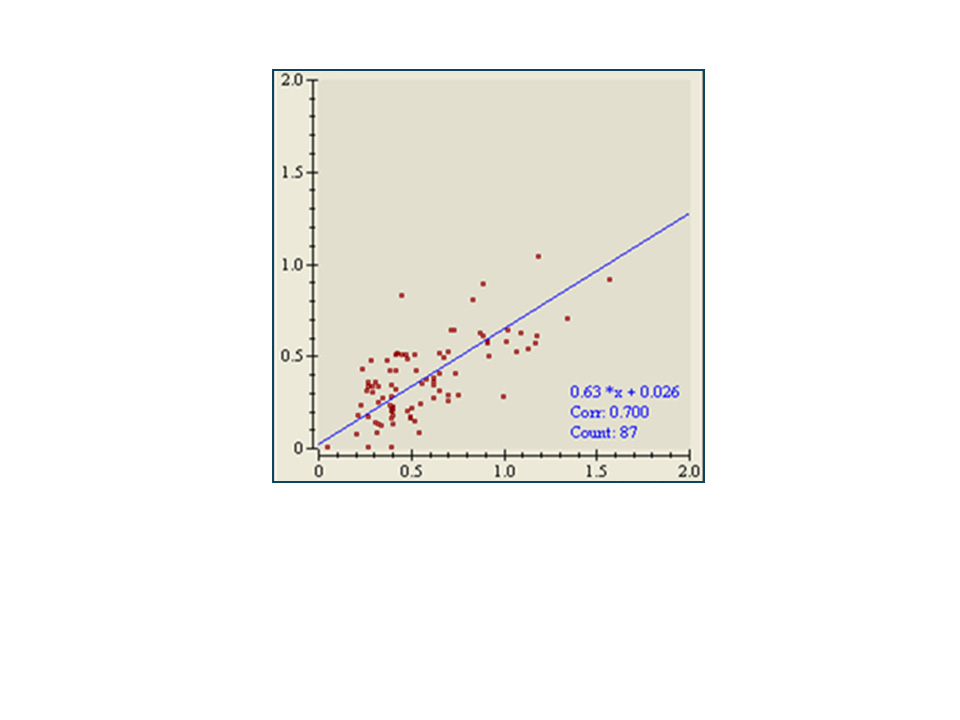

Supplement: Supplementary file 1 [file ijms-26-07924-s001.zip › Figure S1 def .jpg]

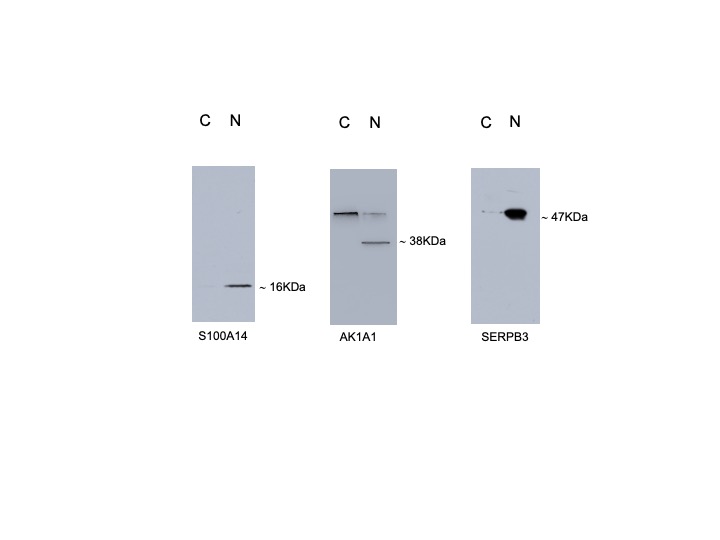

Supplement: Supplementary file 1 [file ijms-26-07924-s001.zip › Figure S2.jpeg]
